# A prospective study of grapefruit and grapefruit juice intake and breast cancer risk

**DOI:** 10.1038/sj.bjc.6604105

**Published:** 2007-11-20

**Authors:** E H Kim, S E Hankinson, A H Eliassen, W C Willett

**Affiliations:** 1 Harvard School of Public Health, Boston, MA 02115, USA; 2 Channing Laboratory, Department of Medicine, Harvard Medical School & Brigham and Women's Hospital, Boston, MA 02115, USA

**Sir**,

In a recent interesting study by Monroe *et al* (2007), grapefruit intake was associated with an increase in breast cancer risk, and they hypothesised that this might be mediated by an effect on endogenous oestrogen levels. However, the researchers were unable to examine grapefruit juice intake. Therefore, we examined grapefruit and grapefruit juice intake and breast cancer risk in the Nurses' Health Study. Briefly, the Nurses' Health Study is a prospective cohort consisting of women aged 30–55 years in 1976 (Kim *et al* 2006). Medical and lifestyle information was obtained with general follow-up questionnaires every 2 years and with semi quantitative food frequency questionnaires that included intakes of grapefruit and grapefruit juice in 1984, 1986, 1990, 1994, and 1998. In both age-adjusted (not shown) and multivariate analyses adjusted for standard breast cancer risk factors, we found no overall association with either grapefruit or grapefruit juice intake and breast cancer risk among all women in the cohort, and among postmenopausal women only (Table 1). Furthermore, our results did not change once additional covariates – alcohol, saturated fat, dietary fibre, and soluble fibre – included by Monroe *et al* were added to our models.

Stratification by BMI did not alter the breast cancer risk with either grapefruit or grapefruit juice intake. However, stratification by hormone therapy showed a significant decrease in risk of breast cancer with greater intake of grapefruit in women who never used hormone therapy (multivariate RR comparing ¼ grapefruit or more per day to none=0.78, 95% CI, 0.59–1.04, *P* trend=0.03). This is contrary to the findings of Monroe *et al*, who observed a significant increase in risk of breast cancer with greater consumption of grapefruit in this subgroup.

Furthermore, the association between grapefruit (not grapefruit juice) intake and breast cancer risk differed significantly by oestrogen and progesterone receptor status of the tumours. No association was observed in women with oestrogen and progesterone receptor positive cancers. However, in women with oestrogen and progesterone receptor negative cancers, there was a significant decrease in breast cancer risk with increased consumption of grapefruit (multivariate RR comparing ¼ grapefruit or more per day to none=0.60, 95% CI, 0.37–0.98, *P* trend=0.03).

We also examined cross-sectionally the relationship between consumption of grapefruit and grapefruit juice and plasma levels of oestrogens among 701 postmenopausal women not using hormone replacement. No significant correlation was observed (grapefruit, grapefruit juice) for plasma oestradiol (*r*=0.02, −0.04), oestrone (*r*=0.00, −0.02), or oestrone sulphate (0.09, 0.01).

Our findings do not support an adverse effect of consumption of grapefruit or grapefruit juice on risk of breast cancer or on endogenous hormone levels.

## Figures and Tables

**Table 1:** Multivariate relative risks of breast cancer incidence between 1984 and 2002 by cumulatively averaged grapefruit and grapefruit juice intake

*All women 77 050 subjects (total cases*=*4315)*					
*Grapefruit (per day)*	*0*	>*0–1*/*8*	⩾*1*/*8–1*/*4*	⩾*1*/*4*	*P Trend*
Cases	969	2576	539	231	
Multivariate RR (95% CI)	1.00	1.02 (0.94, 1.10)	0.96 (0.86, 1.07)	1.00 (0.86, 1.15)	0.5
*Grapefruit juice (small glass per day)*	*0*	>*0–1*/*4*	⩾*1*/*4*–*1*/*2*	⩾*1*/*2*	*P Trend*
Cases	2335	1571	265	144	
Multivariate RR (95% CI)	1.00	0.96 (0.89, 1.02)	1.01 (0.89, 1.15)	0.95 (0.80, 1.13)	0.52
					
*Postmenopausal women only 73 411 subjects (total cases*=*3570)*					
*Grapefruit (per day)*	*0*	>*0–1*/*8*	⩾*1*/*8–1*/*4*	⩾*1*/*4*	*P Trend*
Cases	732	2169	469	200	
Multivariate RR (95% CI)	1.00	1.03 (0.95, 1.12)	0.97 (0.86, 1.09)	0.97 (0.83, 1.14)	0.34
*Grapefruit juice (small glass per day)*	*0*	>*0–1*/*4*	⩾*1*/*4–1*/*2*	⩾*1*/*2*	*P Trend*
Cases	1853	1360	228	129	
Multivariate RR (95% CI)	1.00	0.99 (0.92, 1.06)	1.04 (0.90, 1.19)	1.02 (0.85, 1.22)	0.95

Values are adjusted for energy, age, time period, height, parity, age at first birth, weight change since age 18 years, body mass index at age 18 years, age at menopause, use of hormone replacement therapy, family history of breast cancer, benign, breast disease, age at menarche and physical activity.
